# Supplementary material for: NLRP6 Induces Lung Injury and Inflammation Early in Brucella and Influenza Coinfection
Source: J Pers Med. 2022 Dec 14;12(12):2063. doi: 10.3390/jpm12122063 (PMC9785007; doi:10.3390/jpm12122063)
Supplement: Supplementary file 1 [file jpm-12-02063-s001.zip › Supplementary_table.pdf]

Supplementary Material

KEY RESOURCES TABLE

REAGENT or RESOURCE	SOURCE	IDENTIFIER
**Bacterial and virus strains**		
Influenza A/PR/8/1934 (H1N1)	The paper	N/A
Brucella *Suis* strain S2	AOLONG	Cat#CVCC70502
**Chemicals, peptides, and recombinant proteins**		
TPCK-treated trypsin	Sigma	Cat#4370285
BBL™ Trypticase™ Soy Agar	BD	Cat#211043
BBL™ Trypticase™ Soy Broth	BD	Cat#257107
Brucella selective supplement	CBMORE	Cat#CS1901
Brucella Medium Base	CBMORE	Cat#CB001138
RNAprep Pure Tissue Kit	Tiangen	Cat#DP431
First-strand cDNA Synthesis Supermix	Transcript	Cat#AT301
PowerUp™ SYBR™ Green Master Mix	Thermo Fisher	Cat#A25741
BCA Protein Assay Kit	Genstar	Cat#E162-05
Direct-load Color Prestained Protein Marker	Genstar	Cat#M221-10
HRP-conjugated GAPDH Monoclonal antibody	Proteintech	Cat#HRP-60004
GSDMD Polyclonal antibody	Proteintech	Cat#20770-1-AP
NLRP6 Polyclonal antibody	Immunoway	Cat#YN4671
pre-cast polyacrylamide BisTris gel	Solarbio	N/A
Goat Anti-Rabbit IgG H&L (HRP)	Abcam	Cat#ab6721
Mouse TNF-α(Tumor Necrosis Factor Alpha) ELISA Kit	elabscience	Cat#E-EL-M3063
Mouse IL-18(Interleukin 18) ELISA Kit	elabscience	Cat#E-EL-M0730c
Mouse IL-1β(Interleukin 1 Beta) ELISA Kit	elabscience	Cat#E-EL-M0037c
**Experimental models: Cell lines**		
Canine: MDCK (NBL-2) cells	ATCC	Cat#CCL-34
**Experimental models: Organisms/strains**		
Mouse: C57BL/6N	This paper	N/A
Mouse: NLRP6$^{-/-}$C57BL/6N	This paper	N/A
**Oligonucleotides**		
Primers	Table S1	N/A

**Software and algorithms**		
SPSS 22	IBM	https://www.ibm.com/cn
GraphPad 9.0	OriginLab	https://www.graphpad.com/scientific-software/prism

Table S1: The primers used for q-PCR.

Gene	Forward primer	Reverse primer
Influenza M1	5'-aagaccaatcctgtcacctctg-3'	5'-caaaacgtctacgctgcagtcc-3'
GAPDH	5‘-aggtcggtgtgaacggatttg-3’	5‘- tgtagaccatgtagttgaggtca-3’
NLRP6	5'-agtttagcccagaaagga-3'	5'-ccagtgtagccataagcag-3'
GSDMD	5'-gaattcatgccatcggcctttgag-3'	5'-ggatccatctgacaggagactgagc-3'
IL-1β	5'-gccagtgaaatgatggcttatt-3'	5'-aggagcacttcatctgtttagg-3'
IL-18	5'-actgtacaaccgcagtaatacgc-3'	5'-agtgaacattacagatttatccc-3'
